# The Role of Cerium Valence in the Conversion Temperature of H_2_Ti_3_O_7_ Nanoribbons to TiO_2_-B and Anatase Nanoribbons, and Further to Rutile

**DOI:** 10.3390/molecules28155838

**Published:** 2023-08-03

**Authors:** Polona Umek, Michael Dürrschnabel, Leopoldo Molina-Luna, Srečo Škapin, Romana Cerc Korošec, Carla Bittencourt

**Affiliations:** 1 Jožef Stefan Institute, Jamova Cesta 39, 1000 Ljubljana, Slovenia; sreco.skapin@ijs.si; 2 Karlsruhe Institute of Technology, P.O. Box 6980, 706049 Karlsruhe, Germany; michael.duerrschnabel@kit.edu; 3 Department of Materials and Earth Sciences, Technische Universität Darmstadt, Peter-Grünberg-Strasse 2, 64287 Darmstadt, Germany; molina@aem.tu-darmstadt.de; 4 Faculty of Chemistry and Chemical Technology, University of Ljubljana, Večna Pot 113, 1000 Ljubljana, Slovenia; romana.cerc-korosec@fkkt.uni-lj.si; 5 Chimie des Interactions Plasma-Surface (ChIPS), Research Institute for Materials Science and Engineering, University of Mons, 7000 Mons, Belgium

**Keywords:** TiO_2_, TiO_2_-B, anatase, CeO_2_, impregnation, ion exchange, transformation, calcination, CeO_2_-TiO_2_, mixed oxides

## Abstract

CeO_2_-TiO_2_ is an important mixed oxide due to its catalytic properties, particularly in heterogeneous photocatalysis. This study presents a straightforward method to obtain 1D TiO_2_ nanostructures decorated with CeO_2_ nanoparticles at the surface. As the precursor, we used H_2_Ti_3_O_7_ nanoribbons prepared from sodium titanate nanoribbons by ion exchange. Two cerium sources with an oxidation state of +3 and +4 were used to obtain mixed oxides. HAADF–STEM mapping of the Ce^4+^-modified nanoribbons revealed a thin continuous layer at the surface of the H_2_Ti_3_O_7_ nanoribbons, while Ce^3+^ cerium ions intercalated partially between the titanate layers. The phase composition and morphology changes were monitored during calcination between 620 °C and 960 °C. Thermal treatment led to the formation of CeO_2_ nanoparticles on the surface of the TiO_2_ nanoribbons, whose size increased with the calcination temperature. The use of Ce^4+^ raised the temperature required for converting H_2_Ti_3_O_7_ to TiO_2_-B by approximately 200 °C, and the temperature for the formation of anatase. For the Ce^3+^ batch, the presence of cerium inhibited the conversion to rutile. Analysis of cerium oxidation states revealed the existence of both +4 and +3 in all calcined samples, regardless of the initial cerium oxidation state.

## 1. Introduction

Titanium dioxide (TiO_2_), owing to its unique combination of properties, high chemical stability, and low cost, is one of the most investigated materials used in a wide range of applications [1,2,3]; due to its high refractive index, opacity, and brightness, it has been widely used as a white pigment in paints, coatings, and plastics [4]. It is a wide-gap semiconducting material with Eg~3.2 eV exhibiting photocatalytic activity. This property makes it useful in air and water purification, self-cleaning surfaces, and even hydrogen fuel production [5]. Due to its biocompatibility, it has been used as material for prostheses in dental, orthopedic, and osteosynthesis applications [6,7]. 

Several approaches were suggested to tune the TiO_2_ properties, including particle size, particle shape and surface modification, and doping, to mention a few [8,9]. In the case of particle size and shape modification, several synthetic approaches were proposed for the preparation of nanosized TiO_2_, including sol-gel, hydrothermal and solvothermal synthesis, microemulsion [10], and co-precipitation methods [11,12,13]. One-dimensional (1D) TiO_2_ nanostructures, such as nanotubes, nanowires, and nanoribbons, can be prepared from 1D hydrogen titanates (H_2_Ti_n_O_2n+1_) by thermal treatment in air [14,15,16]. Each method has its advantages and disadvantages, and the choice of method will depend on the specific application and desired properties of the nanosized TiO_2_ material. Doping of TiO_2_ with various transition metals or lanthanide ions can improve its properties by increasing its visible light absorption, enhancing its photocatalytic activity, and improving its charge separation efficiency [1,17]. Among the rare earth elements, cerium has received attention as cerium doping enhances optical absorption in the visible region and inhibits the recombination rate of photo-generated carriers [18,19,20,21,22]. Additionally, doping with lanthanide ions can modify the band structure of TiO_2_, improving its electronic and optical properties, and as a result, affecting its catalytic properties [23,24].

1D hydrogen trititanate nanostructures, i.e., H_2_Ti_3_O_7_, have been exploited as an attractive precursor for the preparation of 1D TiO_2_ nanostructures for three main reasons: (i) these materials are thermally unstable, and upon heating, they readily transform to TiO_2_ [25,26,27,28], (ii) they can be prepared in several 1D morphologies, such as nanotubes (HTiNTs) [29,30,31,32], nanowires [33,34,35], and nanoribbons (HTiNRs) [15,36], and (iii) low cost and facile synthesis routes. Three TiO_2_ polymorphs, TiO_2_-B, anatase, and rutile, can be obtained by the thermal treatment of H_2_Ti_3_O_7_ in air [15,16,37]. At elevated temperatures, anatase irreversibly transforms to rutile [38]. The fragmentation of the nanoribbons accompanies this transformation. On the other hand, the transformations from H_2_Ti_3_O_7_ to TiO_2_-B and further to anatase are topotactic reactions as the morphology is preserved due to structural similarities between these three structures [25,39,40]. The transformation of H_2_Ti_3_O_7_ to TiO_2_-B is not a direct one; it occurs in several dehydration steps [25,41]. The transformation temperatures strongly depend on the starting H_2_Ti_3_O_7_ morphology. For instance, for nanoribbons with diameters up to 300 nm and lengths ranging from 500 nm to several microns, the conversion temperature for TiO_2_-B is approximately 400 °C [15,42]. For nanotubes that are hollow structures with average outer diameters of 12 nm and an inner between 5 and 8 nm, less energy is required. They transform already at 250 °C [32,43]. By doping, in small quantities, the conversion temperatures can be significantly altered, as in the case of HTiNRs doped with 1.5 wt.% of Mn^2+^, where rutile was reported to appear at 700 °C [36].

There have been only a few reports on doping 1D titanate [44,45,46] and TiO_2_ nanostructures [47] with cerium. In the report by Viana et al. [44], sodium titanate nanotubes were used as a support for decoration with CeO_2_ particles; (NH_4_)_2_Ce(NO_3_)_6_ was used as a cerium source. By an ion-exchange reaction, Ce^4+^ ions replaced Na^+^ ions between the titanate layers, and CeO_2_ nanoparticles grew on the surface of the nanotubes. It was shown that this modification shifted the adsorption band toward the visible range. Marques et al. [45] evaluated the critical concentration of Ce^4+^ in the aqueous solution at which CeO_2_ nanoparticles started forming in the titanate nanotubes’ surface. These authors showed the presence of cerium in oxidation states of +4 and +3; the latter was formed via reduction reactions in an aqueous solution of the Ce^4+^ precursor. The Ce^3+^ ions also led to the successful ion exchange of sodium ions [46]. CeO_2_ coating of TiO_2_ nanotubes obtained by an anodization process increased the bioactivity of titania, as reported in [47]. 

We prepared two sets of H_2_Ti_3_O_7_ nanoribbons (HTiNRs): (i) in the first one, the surface of HTiNRs was coated with a thin continuous cerium-containing layer, while in the second, (ii) cerium ions intercalated between the titanate layers in a low amount via an ion exchange reaction. For impregnation/intercalation of HTiNRs, two cerium salts, as sources of Ce^4+^ and Ce^3+^, respectively, (Ce(SO_4_)_2_·4H_2_O and Ce(NO_3_)_3_·6H_2_O), were chosen to evaluate the effects of the starting cerium valence on surface impregnation and/or intercalation between titanate layers. The impact of cerium valence (+4 and +3) on modifying H_2_Ti_3_O_7_ nanoribbons and in converting H_2_Ti_3_O_7_ to TiO_2_-B, and further to anatase and rutile, were investigated. H_2_Ti_3_O_7_ nanoribbons were selected as a precursor of TiO_2_ nanoribbons for transformation conducted by calcination for three reasons: (i) a 1D shape that is preserved until anatase formation, (ii) a transformation pathway going through three TiO_2_ crystalographic phases, and (iii) a layered structure enabling cation intercalation. The difference in the oxidation state of the initial cerium ions was found to be crucial for the HTiNRs modification; in the case of Ce^4+^, a thin continuous cerium-containing layer at the surface of the nanoribbons was formed, while for the Ce^3+^, cerium ions via an ion exchange reaction intercalated between the titanate layers in a small amount. The transformation of cerium-modified H_2_Ti_3_O_7_ nanoribbons to TiO_2_-B, and then to anatase and rutile upon increasing the calcination temperature, between 400 °C and 960 °C, was monitored. The temperature required for the formation of TiO_2_-B for both ions was higher than for the non-cerium-modified nanoribbons. The formation of CeO_2_ nanoparticles on the surface of 1D TiO_2_ nanostructures in the same temperature range was observed. Cerium in oxidation states of +4 and +3 were observed in all cerium-containing samples, regardless of the starting oxidation state of cerium. Interestingly, the presence of cerium in anatase nanoribbons prepared with the Ce^3+^ increased the temperature for the conversion to rutile.

## 2. Results and Discussion

Hydrogen titanate nanoribbons (HTiNRs), with an average width of 30–300 nm and length of 0.5–10 μm, were used as a precursor for surface impregnation/intercalation with cerium and further transformation to CeO_2_-TiO_2_ NR composites via calcination in air. A characteristic scanning electron microscopy (SEM) image of obtained HTiNRs revealed a high nanoribbon content with a smooth surface (Figure S1). Their powder X-ray diffraction (XRD) pattern (Figure S2, bottom panel) corresponds to the pattern of H_2_Ti_3_O_7_ as reported in the literature [15,27,37].

### 2.1. Characterization of H_2_Ti_3_O_7_ Nanoribbons Impregnated/Intercalated with Cerium

**Figure 1:**
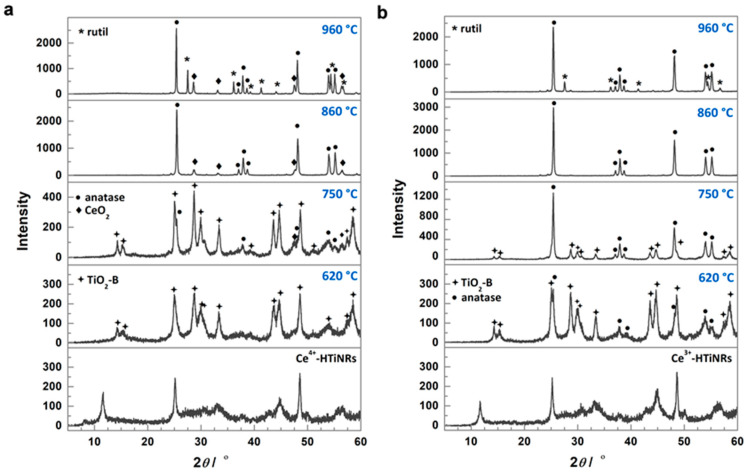
Impact of cerium oxidation state in the starting cerium source on the transformation temperature of H_2_Ti_3_O_7_ nanoribbons. XRD patterns of the precursor materials Ce^4+^-HTiNRs (**a**) and Ce^3+^-HTiNRs (**b**), and products obtained by calcination in air at 620, 750, 860, and 960 °C.

Two cerium salts sources Ce(SO_4_)_2_·4H_2_O and Ce(NO_3_)_3_·6H_2_O were used to prepare two sets of HTiNRs impregnated/intercalated with cerium, Ce^4+^-HTiNRs, and Ce^3+^-HTiNRs, respectively. The impregnation of HTiNRs with the Ce^4+^ precursor affected the color of the nanoribbons, which changed from white to pale yellow. While the change in color of Ce^3+^-HTiNRs was not visible to our eyes. The powder X-ray diffraction patterns (XRDs) (Figure 1a,b, bottom panels) matched those reported for layered trititanate, i.e., H_2_Ti_3_O_7_ (Figure S2, bottom panel) [15,27,37].

**Figure 2:**
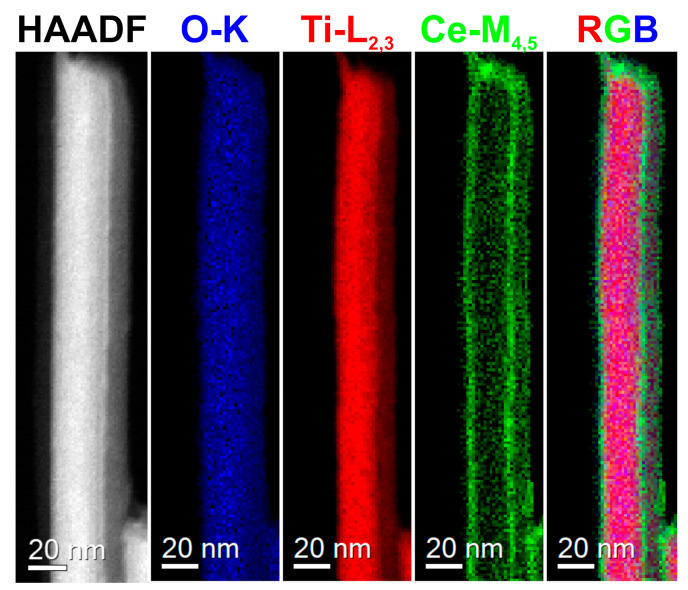
HAADF–STEM image of an individual Ce^4+^-HTiNRs with corresponding elemental maps of Ti, O, Ce (EELS), and an RGB image.

The peak at ~10° in the XRDs of the titanate samples (Figure 1 and Figure S2), i.e., HTiNRs, Ce^3+^-HTiNRs, and Ce^4+^-HTiNRs, arises from the layered trititanate structure and gives information about the interlayer distance [48]. For Ce^3+^-HTiNRs, a shift in this peak from 10.54° to 10.68° was observed (Figure S3), indicating the contraction of the interlayer distance. Due to the bigger size of cerium (III) than the hydronium ion, the interlayer distance was expected to increase [49]. Since cerium (III) is a trivalent ion and hydronium ion is monovalent, one Ce^3+^ changes to three hydronium ions. Therefore, it might be assumed that only a smaller amount of hydronium ions was exchanged with Ce^3+^ ions; the exchange of hydronium ions with cerium (III) ions resulted in the removal of the interlayer water and the formation of vacancies, which promoted the strengthening of the Ti-O bonds, resulting in contraction of the interlayer distance [45]. Conversely, no shift in the peak ~10° for Ce^4+^-HTiNRs was observed, which might be associated with the formation of Ce^4+^ polynuclear species [50,51,52,53], which are too large to intercalate between the titanate layers. This is supported by scanning transmission electron microscopy–electron energy loss spectroscopy (STEM–EELS) analysis (Figure 2). A comparison of the elemental maps (Ti, O, and Ce) reveals that the nanoribbon surface is covered with a continuous ~4 nm thick cerium layer. As expected, the impregnation of HTiNRs with Ce^4+^ and Ce^3+^ did not affect either the material’s morphology or the surface of the NRs, which appears smooth, as revealed by SEM investigation (Figure S4).

The amount of cerium salts used for wet impregnation was calculated to give around 5 wt.% of cerium in the final product, that is, TiO_2_ NRs. The cerium content in Ce^4+^-HTiNRs, determined with energy dispersive X-ray spectroscopy (EDXS) analysis, is 3.4 wt.% and 0.3 wt.% for Ce^4+^-HTiNRs and Ce^3+^-HTiNR, respectively (Table 1). 

Results of thermo-gravimetric analysis (TGA) of both Ce-containing HTiNR samples (Figure S5) are similar to the TGA curve of pure HTiNRs [15]. However, a slightly higher weight loss (1.1 wt.%) was observed for the HTiNRs impregnated with Ce^4+^, which can be explained by a higher cerium content (Table 1) [51,52,53].

### 2.2. Conversion of H_2_Ti_3_O_7_ Nanoribbons to TiO_2_ by Thermal Treatment in Air

Based on a preliminary study, the calcination temperatures were set to 620 °C, 750 °C, 860 °C, and 960 °C. In addition, a batch of samples obtained by calcination of pristine HTiNRs for the comparison of phase composition and morphology was prepared. Sample labels, calcination temperatures, phase compositions, cerium contents, cerium oxidation states, and band gap values are listed in Table 1. 

#### 2.2.1. Cerium Content

The cerium content of the calcined samples, obtained with EDXS analysis, is given in Table 1. At the final calcination temperature of 960 °C, the cerium content is 3.7 and 0.4 wt.% for Ce^4+^ and Ce^3+^ products, respectively. Small amounts of sulfur were detected in the samples obtained by calcinating the Ce^4+^-HTiNRs. The sulfate group in the cerium precursor fully decomposed only above 800 °C. In comparison, the decomposition of the nitrate group of the source Ce(NO_3_)_3_·6H_2_O occurs before reaching 300 °C (Figures S6 and S7).

#### 2.2.2. Structural Determination of TiO_2_ Polymorphs

The XRD pattern of the pristine HTiNRs (H_2_Ti_3_O_7_ NRs) sample calcined at 620 °C shows the presence of the anatase phase (ICCD card no. 86-1157) and traces of TiO_2_-B (ICCD card no. 35-0088); the transformation to anatase was completed at 860 °C. With an increased calcination temperature to 960 °C, in addition to anatase related peaks, new peaks corresponding to rutile (ICCD card no. 89-0555) appeared (Figure S2). For pristine HTiNRs, the transformation to TiO_2_-B was reported to occur typically at 400 °C [15,42]. While for the complete conversion of Ce^4+^-HTiNRs to TiO_2_-B, a higher temperature (620 °C) was required (Figure 1a). As the temperature was raised to 750 °C, TiO_2_-B partly converted to anatase, and new peaks corresponding to CeO_2_ appeared (ICCD card no. 81-0792). Transformation to anatase was completed at 860 °C; all peaks in the Ce^4+^-860 °C diffractogram corresponded to anatase and CeO_2_. With a further increase in the calcination temperature to 960 °C, anatase partially converted to rutile. In the case of the Ce^3+^-HTiNRs precursor (Figure 1b), at 620 °C, the main phase was TiO_2_-B with traces of anatase. At 750 °C, almost all TiO_2_-B is converted to anatase. Similar to the Ce^4+^-HTiNR batch, the transformation to anatase was completed at 860 °C. In Ce^3+^-HTiNRs, anatase was partially converted to rutile as the temperature was raised to 960 °C (Figure 1b).

Next, from the intensities of the (101) anatase and (110) rutile peaks (Figure 1a,b and Figure S2), positioned at ~25.3° and 27.4°, respectively, for the samples calcined at 960 °C the amount of rutile was calculated using the equation suggested by Spurr and Mayers [54] (Table 2). In p-960 °C, rutile was already the majority phase (60 wt.%), while in Ce^4+^-960 °C and Ce^3+^-960 °C, the amount of rutile was 31% and 17%, respectively. This might suggest that the cerium source and location of cerium ions in anatase nanoribbons may play an essential role in inhibiting the transformation to rutile. Therefore, regardless of the cerium source and content, the presence of cerium in HTiNRs, i.e., Ce^3+^-HTiNRs and Ce^4+^-HTiNRs (Table 1), significantly raised the temperature for both transformations, TiO_2_-B to anatase and anatase to rutile, when compared to the pristine HTiNRs (Figure S2) [15,42,55], or as reported to Mn^2+^-doped HTiNRs [36], where rutile had already appeared below 600 °C.

In addition, the size of CeO_2_ nanoparticles was calculated from the (111) CeO_2_ peak positioned at 28.7° (ICDD card no. 81-0792) using the Scherer formula (d = 0.9 λ/B cos θ; λ = 1.54 Å, where B is the full width at half maximum and is the Bragg angle). The calculated values are 22 nm and 33 nm for Ce^4+^-860 °C and Ce^4+^-960 °C, respectively.

#### 2.2.3. Changes in the Nanoribbon Morphology and Formation of CeO_2_ Nanoparticles

Figure S8 summarizes the impact of the calcination temperature on changes in the nanoribbons’ shape for samples prepared by calcination of Ce^4+^-HTiNRs. As expected, the nanoribbon morphology was intact until 750 °C. At 860 °C, the diameter along individual nanoribbons was no longer uniform. At some places along the nanoribbons, thicker areas appeared, and the nanoribbon edges became rounder due to the sintering effect. At 960 °C, the observed elongated structures were much thicker (Figure S8d) than the starting nanoribbons [42]; it looked as if the elongated thickened parts, originating from the nanoribbons, formed a kind of three-dimensional network. In addition, as expected, the fragmentation of nanoribbons, caused by the anatase to rutile transformation, was more severe for p-960 °C (Figure S9) due to a higher mass fraction of rutile compared to Ce^4+^-960 °C and Ce^3+^-960 °C (Table 2). Interestingly, the nanoribbon morphology was less affected in Ce^3+^-960 °C than in Ce^4+^-960 °C, which is associated with a lower amount of rutile in the former (Table 2), suggesting that cerium species in Ce^3+^-960 °C inhibited the conversion to rutile. Similar findings were observed when CeO_2_ was added to the CuO-TiO_2_ system [56].

**Figure 3:**
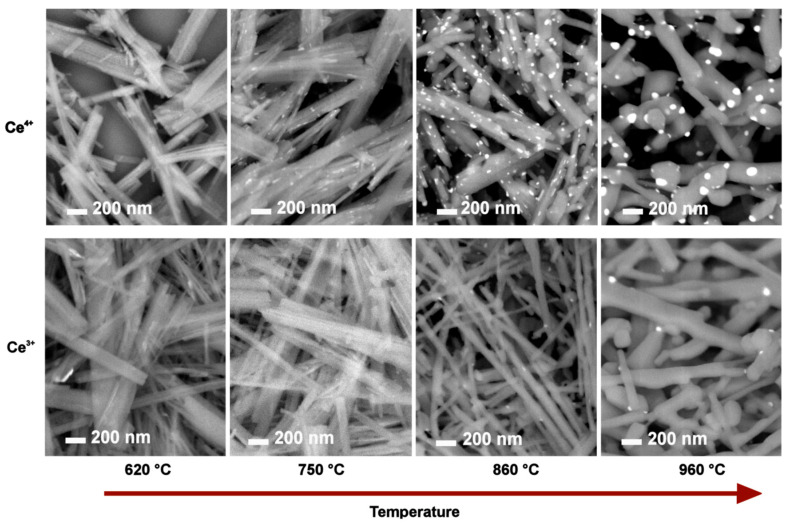
Evolution of CeO_2_ nanoparticles on the surface of TiO_2_ nanoribbons and fragmentation of nanoribbon morphology with increasing calcination temperatures for Ce^4+^-HTiNRs (**top**) and Ce^3+^-HTiNRs (**bottom**), which were calcined at 620 °C, 750 °C, 860 °C, and 960 °C. SEM images using a back-scattered electron detector were taken at the same magnification. Areas with CeO_2_ nanoparticles in the images appear brighter.

Upon calcination, CeO_2_ nanoparticles were observed on the surface of the TiO_2_ nanoribbons (NPs) (Figure 3); for the Ce^4+^ batch, they were observed (in SEM images) at 750 °C, while for the Ce^3+^ batch, they were observed at 860 °C. The difference in the temperature at which CeO_2_ NPs start to be observed can be associated with the cerium content, which was much lower for the Ce^3+^ batch (Table 1), and the fact that cerium ions in Ce^3+^-HTiNTs intercalated between the titanate layers needed more to migrate to the surface. Upon a higher calcination temperature, cerium atoms between the layers diffuse toward the surface, where they segregate, forming CeO_2_ NPs [56]. Therefore, we decided to investigate the sample calcined at the lowest temperature (Ce^4+^ batch) in more detail. STEM–EDX elemental mapping of Ce^4+^-620 °C revealed a small number of cerium-containing nanoparticles with diameters below 20 mm on the surface of the nanoribbons (Figure S11).

With the increasing calcination temperature, the CeO_2_ NP size increased. The density distribution of CeO_2_ NPs was higher at the final calcination temperature (960 °C) for the Ce^4+^ sample than for the Ce^3+^ sample due to a higher cerium content (Table 1). The measured diameter of CeO_2_ NPs was between 13 and 35 nm for Ce^4+^-850 °C, while in Ce^4+^-960 °C, their size was between 20 and 70 nm (Figure S10), which agreed with the calculated values from the XRDs. 

#### 2.2.4. Determination of Cerium Oxidation State and the Ratio between Ce^4+^/Ce^3+^

**Figure 4:**
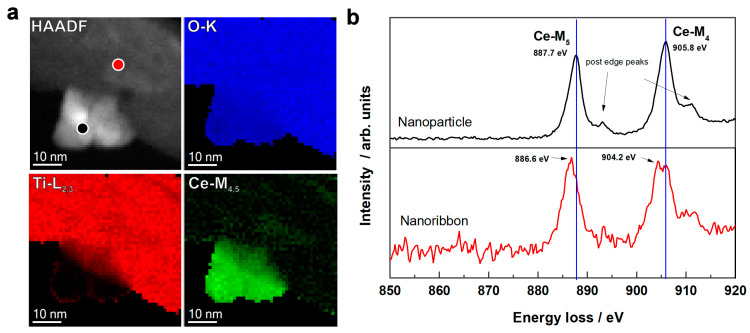
STEM–EELS analysis of sample Ce^4+^-620 °C. (**a**) HAADF image with corresponding elemental maps of O, Ti, and Ce. (**b**) EEL spectra recorded at the nanoribbon surface (red spot in the HAADF image) and a particle attached to the surface of the nanoribbon (black spot in the HAADF image).

CeO_2_ is known for its unique redox chemistry. Oxidation states +3 and +4 steadily exist and can easily switch between each other [57,58]. Therefore, Ce^4+^-620 °C was investigated by STEM–EELS measurements to determine the cerium valence, at a nanoparticle and at a region of a nanoribbon without a particle. (Figure S11). The results are shown in Figure 4; the circles in the high-angle annular dark-field (HAADF) image denote the exact locations from which the electron energy loss (EEL) spectra of the Ce-M_4,5_ were measured (Figure 4a,b). In the EEL spectrum measured over the nanoparticle beside the M_4_ and M_5_ edges, the post-edge peaks associated with Ce^4+^ appeared. The M_4_ and M_5_ edges in the spectrum measured at the nanoribbon shifted to a lower energy loss by 1.6 and 1.1 eV, respectively, which is characteristic of Ce^3+^. Therefore, we can conclude that cerium at the surface of the nanoribbon was a mixture of Ce^4+^ and Ce^3+^, and in the nanoparticle, it is more Ce^4+^. 

**Figure 5:**
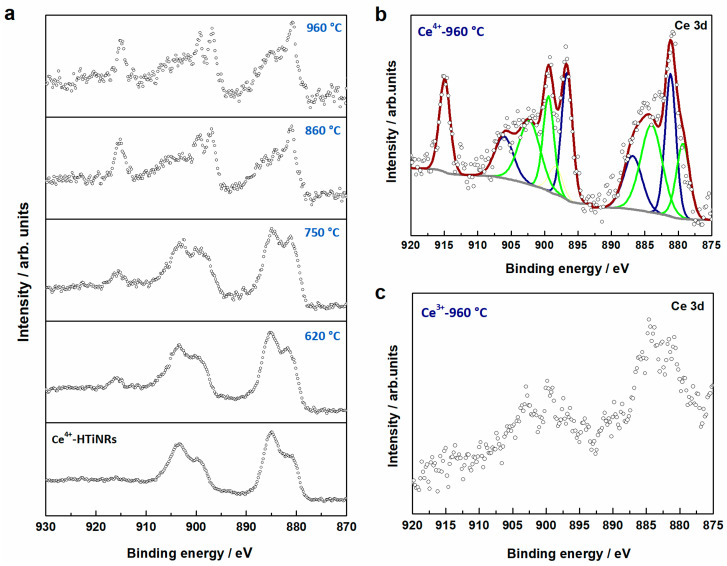
(**a**) Comparison of X-ray photoelectron spectra recorded in the Ce 3d core level region for Ce^4+^-HTiNRs and products obtained after calcination at 620 °C, 750 °C, 860 °C, and 960 °C. (**b**) Result of the peak fitting in the Ce 3d core level X-ray photoelectron spectrum of the sample calcined at 960 °C; green peaks refer to Ce^3+^ and blue ones to Ce^4+^. (**c**) Spectrum of Ce^3+^-960 °C in the Ce 3d core level region.

X-ray photoelectron spectroscopy (XPS) was used to probe a larger sample area to determine the cerium oxidation states and their ratios in the samples derived by the calcination of Ce^4+^-HTiNRs (Table 1 and Figure 5). In all samples obtained from Ce^4+^-HTiNRs, the presence of cerium in oxidation states +4 and +3 was observed, which is not surprising given the facile transformation of Ce^4+^ to Ce^3+^ [57]. In the Ce 3d core level region of the XPS spectra, several characteristic structures of CeO_2_ appeared for annealing at higher temperatures; the main peak is centered at 915 eV and the shoulder at 880 eV of binding energy (Figure 5a,b). The intensity of both structures increased with increasing calcination temperatures, indicating that the amount of CeO_2_ increased, which coincides well with the XRD analysis (Figure 1a and Figure S10). Due to the low cerium content in materials derived from Ce^3+^-HTiNRs (Table 1), evaluation of the ratio of Ce^3+^/Ce^4+^ in these materials was impossible due to the low quality of the spectra; however, the peak structures characteristics for both cerium oxidation states could be observed (Figure 5c).

#### 2.2.5. Optical Band Gap Features

The last column in Table 1 reports the band gap energies of all three sets of samples. The band gap of HTiNRs is 3.45 eV. Surprisingly, the band gap energies of Ce^4+^-HTiNRs and Ce^3+^-HTiNRs are slightly higher, 3.56 and 3.53 eV, respectively. This might be connected with (i) the presence of sodium ions in trace amounts [5] left between the layers after ion exchange (the amount of sodium in HTiNRs determined with EDXS analysis was below the detection limit of 0.1 wt.%), and/or (ii) with the presence of a thin Ce-containing layer on the surface of Ce^4+^-HTiNRs, and (iii) reduced order due to partial exchange of hydronium ions by Ce^3+^ in the case of Ce^3+^-HTiNRs [59]. During thermal treatment in air, the *E_g_* was gradually reduced with increasing calcination temperatures for all three sets of samples. This can be attributed to the formation of TiO_2_-B, anatase, and rutile (for the highest calcination temperature), and to the increasing crystallinity (Figure 1a,b and Figure S1) of the nanoribbons.

Reported band gap energies of crystalline bulk TiO_2_-B and anatase are equal, namely, 3.2 eV [5], and are smaller than those measured for our TiO_2_ samples (Table 1). During the particle size reduction from micro to nano-sized particles, the band gap values of semiconductors are usually increased [60].

## 3. Materials and Methods

### 3.1. Materials Preparation

*Preparation of H_2_Ti_3_O_7_ nanoribbons.* The starting material, H_2_Ti_3_O_7_ nanoribbons (NRs), were prepared according to the method already reported elsewhere [15,61], with the exception that for the preparation of H_2_Ti_3_O_7_ NRs from (Na, H)_2_Ti_3_O_7_ NRs by ion exchange, instead of 0.1 M CH_3_COOH, 0.1 M HCl were used [36].

*Preparation of TiO_2_ NRs decorated with CeO_2_ NPs via calcination in air.* Firstly, a wet impregnation was employed to prepare H_2_Ti_3_O_7_ NRs coated with cerium. As precursors of cerium in oxidation states +4 and +3, Ce(SO_4_)_2_·4H_2_O and Ce(NO_3_)_3_·6H_2_O were used. The amount of cerium precursors was calculated to give approximately 5 wt.% of cerium in TiO_2_ NRs; in brief, 1 g of HTiNRs was dispersed into 120 mL of deionized water, then 50 mL of a water solution of cerium precursor (120 mg of Ce(SO_4_)_2_·4H_2_O and 135 mg of Ce(NO_3_)_3_·6H_2_O) was added dropwise. Prepared mixtures were stirred overnight, then filtrated, and finally dried in air at 100 °C for 10 h. Prepared precursors were labeled Ce^4+^-HTiNRs and Ce^3+^-HTiNRs. In the next step, H_2_Ti_3_O_7_ nanoribbons impregnated with cerium were transformed into TiO_2_ NRs decorated with CeO_2_ nanoparticles (NPs) by calcination in static air; 150 mg of the precursor was weighed in an alumina boat, placed into an oven (Carbolite), and heated to the target temperature (400 °C, 620 °C, 750 °C, 860 °C, and 960 °C) at 1 °C/min. Samples were maintained at the selected temperature for 7 h and then cooled to room temperature. Sample labels, exact reaction conditions, and phase compositions are listed in Table 1.

### 3.2. Materials Characterization

The physical and chemical properties of the prepared materials were analyzed using several experimental techniques. The morphological features were studied using field emission scanning electron microscopes (SEM) (JEOL-7600F, JEOL Tokyo, Japan and Verios G4, ThermoFischer, Waltham, MA, USA) and a transmission electron microscope (HRTEM), JEM 2100 (Jeol Ltd., Akishima City, Japan). Specimens for the SEM analysis were prepared by dispersing a small amount of prepared powder samples in deionized water, and a drop of the dispersion was deposited on a polished surface of an Al sample holder. Before the SEM investigation, a ca. 3 nm thick carbon layer was deposited on the specimens to reduce the charging effect. The crystallinity of the nanoribbons and cerium distribution over the nanoribbons in the samples were investigated with a transmission electron microscope (TEM) Jeol 2100 at 200 keV and a ThermoFisher Talos F200X scanning transmission electron microscope (STEM) equipped with four energy-dispersive X-ray (EDX) detectors and a Gatan Enfinium electron energy-loss (EEL) spectrometer (Gatan, Pleasanton, CA, USA) with DualEELS capability. The microscope was operated at 200 kV, and the energy resolution of the recorded EEL spectra was about 1.2 eV. The convergence and the collection angle for the EELS experiments were 10.5 and 14.1 mrad, respectively. The Ce M5/M4 ratio was determined using the second derivative method described in [62,63]. Specimens for TEM/HAADF–STEM analyses were dispersed ultrasonically in methanol, and a drop of the dispersion was deposited onto a lacy carbon film supported by a copper grid.

Identification of the phase composition of the samples was determined from powder X-ray diffraction (XRD) patterns measured using a D4 Endeavor, Bruker AXS diffractometer, Bruker, Karlsruhe, Germany with Cu Kα radiation (λ = 1.5406 Å) and a Sol-X energy-dispersive detector. Diffractograms were measured in the 2θ angular range with a step size of 0.02° s^−1^ and a collection time of 3 s.

Elemental composition was determined from energy-dispersive X-ray data (EDX) measured using a field emission scanning electron microscope (JEOL 7600F) equipped with an EDX spectrometer elemental analysis system. Specimens for EDX analyses were prepared by pressing powder samples into pellets and placing them on carbon tape on an Al sample holder. The holder with the samples was coated with a thin carbon layer before the analysis.

X-ray spectroscopy (XPS) measurements have been conducted to determine the oxidation states of cerium and titanium. XPS measurements were performed with a VERSAPROBE PHI 5000 (Physical Electronics, Inc., Chanhassen, MN, USA) equipped with a monochromatic Al Kα X-ray source. The energy resolution was 0.7 eV. Specimens for XPS measurements were prepared by pressing the sample powders into pellets. A conductive double-face tape UHV compatible was used to attach the pellet to a sample holder.

Diffuse reflectance UV–Vis spectroscopy was used to obtain the energy gap value of the synthesized samples. The spectra were recorded using a UV–Vis–NIR spectrometer (Shimadzu UV-3600, Tokyo, Japan) equipped with an integrating sphere (ISR-3100, 60 mm) and BaSO_4_ as a reference in the wavelength range of 200 to 800 nm with a 0.5 nm step size and a UV/Vis/NIR spectrometer (PerkinElmer Lambda 950, Hong Kong, China) equipped with a 150-nm sphere. The Kubelka–Munk function was applied to convert the diffuse reflectance into the absorbance [64]. The optical band-gap energy (*E*g) was determined from the wavelength at which the tangent of the absorbance line intersected the abscissa coordinate.

Thermal decomposition of Ce(SO_4_)_2_·4H_2_O was studied using a Mettler Toledo TGA/DSC 1 instrument from room temperature to 900 °C under dynamic airflow (50 mL min^−1^). The heating rate was 10 °C min^−1^. Crystalohydrate with an initial mass of 5.124 mg was placed into a 150 μL alumina crucible. The blank curve was automatically subtracted. Evolved gases were transferred to a mass spectrometer (Pfeiffer Vacuum ThermoStar) via the 75 cm long heated transfer line. For Ce^4+^-HTiNRs, Ce^3+^-HTiNRs, and Ce(SO_4_)_2_·4H_2_O, the same experimental parameters were used for TGA/DSC measurements as previously described. In the case of Ce(NO_3_)_3_·6H_2_O, where we were interested in the DSC signal, a higher initial mass (19.129 mg) was used, while for Ce^4+^-HTiNRs and Ce^3+^-HTiNRs, the initial mass of the sample was around 5 mg. For these three samples, the upper temperature of measurements was 600 °C. The volume of platinum crucibles used was 150 μL.

## 4. Conclusions

The surface of HTiNRs dispersed in a Ce^4+^ aqueous solution was successfully impregnated with a thin 2–4 nm thick continuous cerium-containing layer. When Ce^3+^ was used as the cerium source, ion exchange was more favorable; however, only a small amount of hydronium ions exchanged with the cerium ions. Upon calcination, CeO_2_-TiO_2_ mixed oxide was formed. CeO_2_ nanoparticles formed on the nanoribbons’ surface. For the Ce^4+^ batch, an increase in CeO_2_ nanoparticle size with increasing calcination temperature was observed.

Modification of the composition of HTiNRs with Ce^4+^ and Ce^3+^ significantly raised the temperature for the conversion of H_2_Ti_3_O_7_ to TiO_2_-B, TiO_2_-B to anatase, and anatase to rutile for at least 200 °C. The nanoribbon morphology remained preserved up to 860 °C. Anatase to rutile transformation at 960 °C caused fragmentation of the nanoribbons. In the case of the Ce^3+^ source, at 960 °C, a smaller amount of anatase is converted to rutile. However, the cerium content was about 10 times lower than in the Ce^4+^ sample that calcined at the same temperature, suggesting that cerium atoms occupied positions that prevented the transformation to rutile. 

For the Ce^4+^-batch, the EELS and XPS result showed that Ce^3+^ was present in the impregnated HTiNRs, and that the relative amount of Ce^4+^ increased with an increasing calcination temperature. The presence of cerium in calcined products caused a decrease in the band gap energy.

## Figures and Tables

**Table 1:** Sample labels, transformation conditions, phase composition, cerium content, oxidation state, and band gap values.

Sample Labels	Precursor	Transformation Environment	T (°C)	Phase Composition	Ce CONTENT ^a^ (wt.%)	Ce Oxidation State ^b^	Band Gap (eV)
Ce^4+^-HTiNRs	H_2_Ti_3_O_7_ NRs	DI water ^c^	100	H_2_Ti_3_O_7_	3.4	+3, +4 (80%)	3.56
Ce^4+^-620 °C	Ce^4+^-HTiNRs	static air	620	TiO_2_-B	3.9	+3, +4 (75%)	3.30
Ce^4+^-750 °C	Ce^4+^-HTiNRs	static air	750	TiO_2_-B, anatase	3.9	+3, +4 (70%)	3.17
Ce^4+^-860 °C	Ce^4+^-HTiNRs	static air	860	anatase	3.8	+3, +4 (50%)	3.27
Ce^4+^-960 °C	Ce^4+^-HTiNRs	static air	960	anatase, rutile	3.7	+3, +4 (50%)	3.19
Ce^3+^-HTiNRs	H_2_Ti_3_O_7_ NRs	DI water ^b^	100	H_2_Ti_3_O_7_	0.2	+3, +4	3.53
Ce^3+^-620 °C	Ce^3+^-HTiNRs	static air	620	TiO_2_-B, anatase	0.3	+3, +4	3.40
Ce^3+^-750 °C	Ce^3+^-HTiNRs	static air	750	anatase, traces of TiO_2_-B	0.3	+3, +4	3.36
Ce^3+^-860 °C	Ce^3+^-HTiNRs	static air	860	anatase	0.3	+3, +4	3.34
Ce^3+^-960 °C	Ce^3+^-HTiNRs	static air	960	anatase, rutile	0.4	+3, +4	3.31
HTiNRs	/	/	/	H_2_Ti_3_O_7_	/	/	3.46
^d^ p-620 °C	H_2_Ti_3_O_7_ NRs	static air	620	anatase, traces of TiO_2_-B	/	/	3.31
p-750 °C	H_2_Ti_3_O_7_ NRs	static air	750	anatase	/	/	3.28
p-860 °C	H_2_Ti_3_O_7_ NRs	static air	860	anatase	/	/	3.29
p-960 °C	H_2_Ti_3_O_7_ NRs	static air	960	anatase, rutile	/	/	3.01

^a^ cerium content was determined by EDXS–SEM; ^b^ cerium oxidation state was determined using XPS (for Ce^3+^-modified nanoribbons; due to the small relative amount of cerium, it was impossible to evaluate the ratio between Ce^3+^/Ce^4+^ oxidation states) and ^c^ deionized water. ^d^ p, pristine is used to label samples not doped with cerium.

**Table 2:** The wt.% of rutile in the samples calcined at 960 °C.

Sample	wt.% Rutile
Ce^4+^-960 °C	31
Ce^3+^-960 °C	17
p-960 °C	60

## Data Availability

The data presented in this study are available on request from the corresponding author.

## Supplementary Materials

The following supporting information can be downloaded at: https://www.mdpi.com/article/10.3390/molecules28155838/s1, Figure S1: SEM image of H_2_Ti_3_O_7_ nanoribbons (HTiNRs) used as a precursor for wet impregnation/intercalation with Ce^4+^ and Ce^3+^; Figure S2: XRD of pristine HTiNRs and products calcined at 620, 750, 860, and 960 °C in air; Figure S3: XRD patterns of HTiNRs (top), Ce^4+^-HTiNRs (middle), and Ce^3+^-HTiNRs (bottom) between 7 and 15°. Vertical lines guide the eye to easily observe the (100) peak shift to higher angles for Ce^3+^-HTiNRs; Figure S4: SEM image of HTiNRs impregnated with Ce^4+^ (Ce^4+^-HTiNRs); Figure S5: TGA curves for HTiNRs impregnated with Ce^4+^ (Ce^4+^-HTiNRs) and Ce^3+^ (Ce^3+^-HTiNRs) measured in air; Figure S6: TGA curve of Ce(SO_4_)_2_·4H_2_O measured in air and MS of H_2_O, SO, and SO_2_; Figure S7: TGA and DSC curves of Ce(NO_3_)_3_·6H_2_O measured in air. Figure S8: Breakdown of the nanoribbon morphology with increasing calcination temperature for Ce^4+^-HTiNRs calcined at **a** 620 °C, **b** 750 °C, **c** 860 °C, and **d** 960 °C. TEM images were taken at the same magnification; Figure S9: Collapse of the nanoribbon morphology with increasing calcination temperatures for HTiNRs calcined at 620 °C, 750 °C, 860 °C, and 960 °C. SEM images were taken at the same magnification with a secondary electron detector; Figure S10: CeO_2_ NPs size distribution for Ce^4+^-860 °C and Ce^4+^-960 °C; Figure S11: STEM–EDX elemental mapping of Ce^4+^-620 °C. References [65,66] are cited in the Supplementary Materials.
