# Supplementary material for: The Role of Cerium Valence in the Conversion Temperature of H2Ti3O7 Nanoribbons to TiO2-B and Anatase Nanoribbons, and Further to Rutile
Source: Molecules. 2023 Aug 3;28(15):5838. doi: 10.3390/molecules28155838 (PMC10421187; doi:10.3390/molecules28155838)
Supplement: Supplementary file 1 [file molecules-28-05838-s001.zip › molecules-2527377-supplementary.pdf]

Supplementary

The Role of Cerium Valence on the Conversion Temperature of $H_2Ti_3O_7$ Nanoribbons to TiO_2-B and Anatase Nanoribbons, and Further to Rutile

Polona Umek [1,*], Michael Dürrschnabel [2], Leopoldo Molina-Luna [3], Srečo Škapin [1], Romana Cerc [1], and Carla Bittencourt [5,*]

[1] Jožef Stefan Institute, Jamova cesta 39, 1000 Ljubljana, Slovenia; polona.umek@ijs.si, sreco.skapin@ijs.si
2 Karlsruhe Institute of Technology, P.O. Box 6980, 706049 Karlsruhe, Germany; michael.duerrschnabel@kit.edu
[3] Technische Universität Darmstadt, Peter-Grünberg-Strasse 2, 64287 Darmstadt, Germany; molina@aem.tu-darmstadt.de
[4] Faculty of Chemistry and Chemical Technology, University of Ljubljana, Večna pot 113, 1000 Ljubljana, Slovenia; romana.cerc-korosec@fkkt.uni-lj.si
[5] Chimie des Interactions Plasma-Surface (ChIPS), Research Institute for Materials Science and Engineering, University of Mons, 7000 Mons, Belgium; carla.bittencourt@umons.ac.be

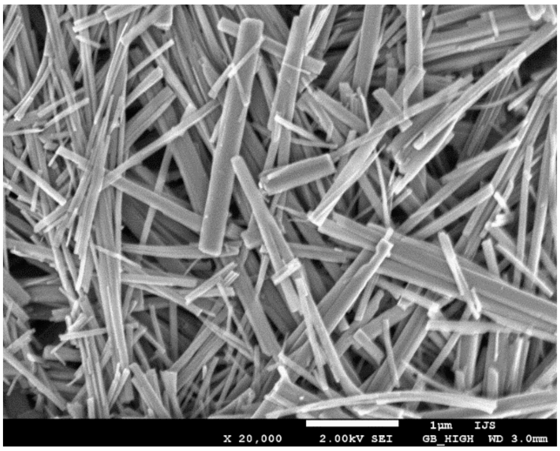

Figure S1. SEM image of $H_2Ti_3O_7$ nanoribbons (HTiNRs) used as a precursor for wet impregnation/intercalation with Ce^{4+} and Ce^{3+}. The width of the nanoribbons (NRs) ranges from 20 to 350 nm, the majority of the NRs have lengths between 1 and 2 µm, although separate NRs in length can reach up to 6 µm.

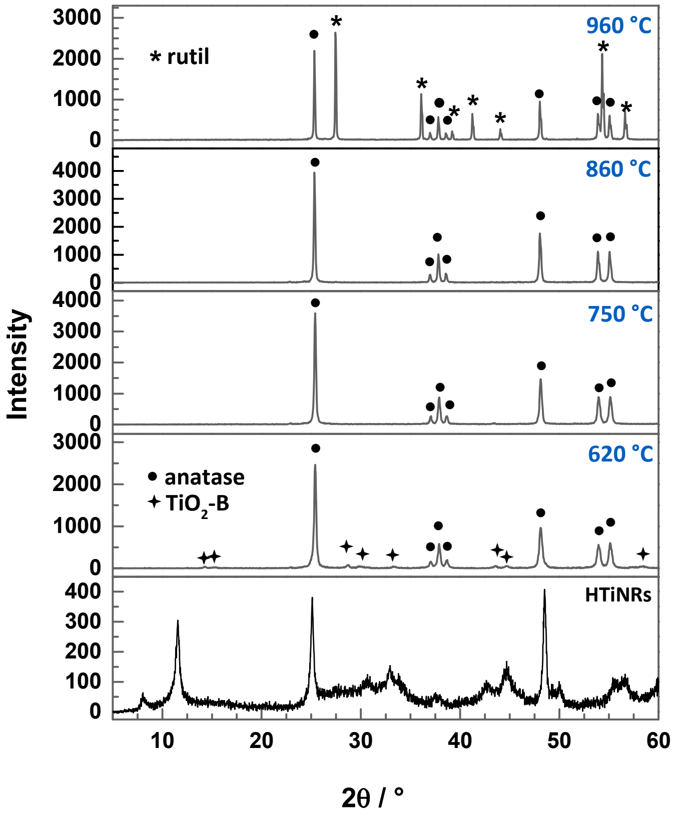

Figure S2. XRD of pristine HTiNRs and products calcined at 620, 750, 860, and 960 °C in air.

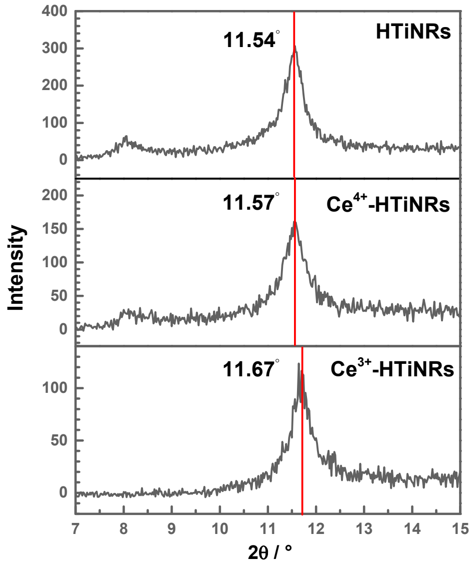

Figure S3. XRD patterns of HTiNRs (top), Ce^{4+}-HTiNRs (middle), and Ce^{3+}-HTiNRs (bottom) between 7 and 15°. Vertical lines guide the eye to easily observe the (100) peak shift to higher angles for Ce^{3+}-HTiNRs.

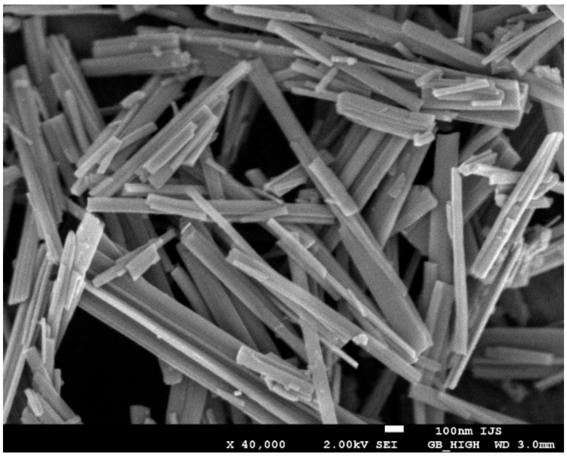

Figure S4. SEM image of HTiNRs impregnated with Ce^{4+} (Ce^{4+}-HTiNRs).

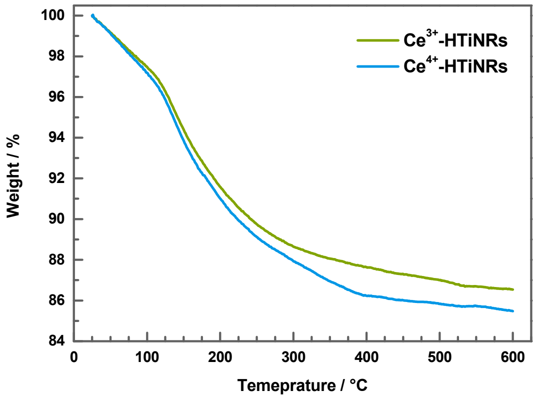

Figure S5. TGA curves for HTiNRs impregnated with Ce^{4+} (Ce^{4+}-HTiNRs) and Ce^{3+} (Ce^{3+}-HTiNRs) measured in air.

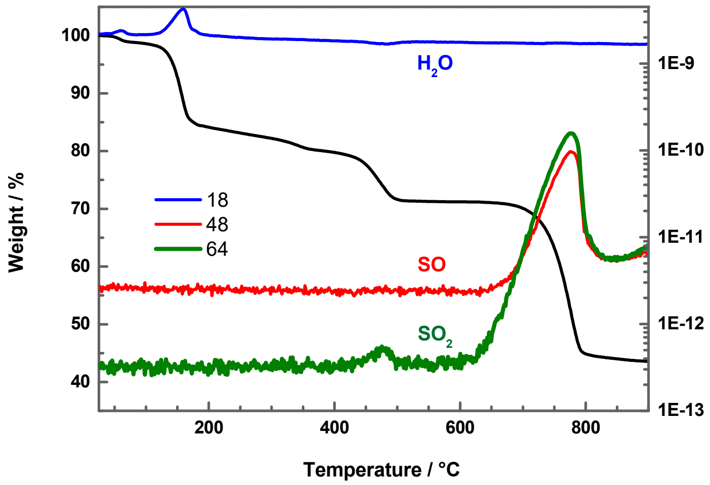

Figure S6. TGA curve of $Ce(SO_4)_2$ $4H_2O$ measured in air and MS of H_2O, SO, and SO_2. Dehydration of crystalline bonded water takes place in one step and is finished at 200 °C. The decomposition of the sulfate group takes place in two steps. The first step starts at about 430 °C, on further heating the second step starts at about 600 °C. Decomposition of the sulfate group is completed above 800 °C, the final product formed is CeO_2 and represents 43.6 wt. % of the starting mass. The theoretical mass loss for $Ce(SO_4)_2$ $2H_2O$ is 57.6 wt. % and agrees well with the observed one [65].

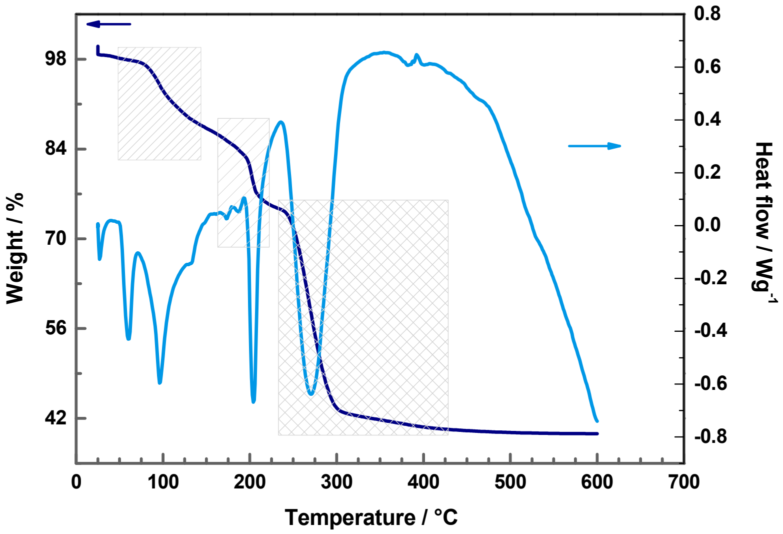

Figure S7. TGA and DSC curves of $Ce(NO_3)_3$ $6H_2O$ measured in air.
Complete dehydration of crystalline water in $Ce_2(NO_3)_3$ $6H_2O$ is finished up to 210 °C, on further heating in the range 230 to 310 °C, decomposition of $Ce_2(NO_3)_3$ takes place accompanied by oxidation of Ce^{3+} to Ce^{4+} resulting in the formation of CeO_2 (39,6 wt. % of the starting mass). Theoretical mass loss for $Ce_2(NO_3)_3$ $6H_2O$ is 60.4 wt. % and agrees well with the observed one [66]

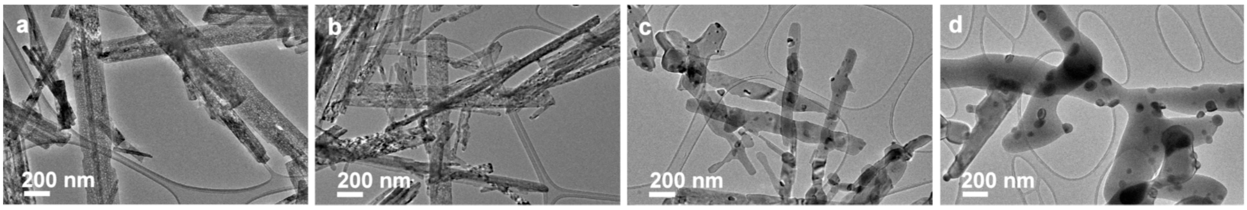

Figure S8. Breakdown of the nanoribbon morphology with increasing calcination temperature for Ce^{4+}-HTiNRs calcined at **a** 620 °C, **b** 750 °C, **c** 860 °C and **d** 960 °C. TEM images were taken at the same magnification.

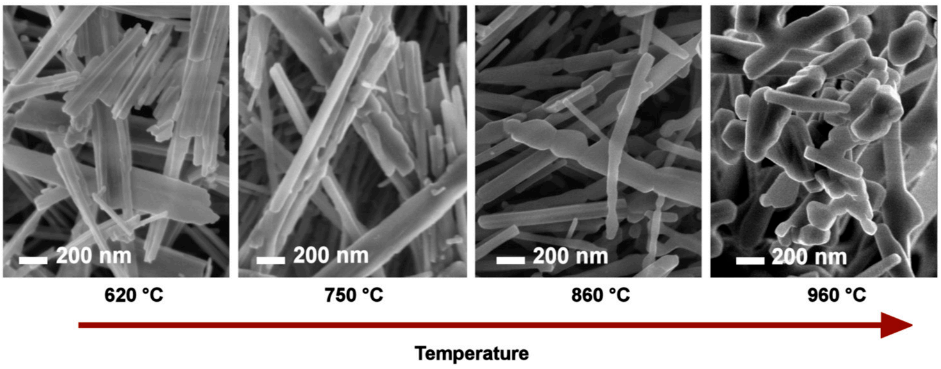

Figure S9. Collapse of the nanoribbon morphology with increasing calcination temperature for HTiNRs calcined at 620 °C, 750 °C, 860 °C, and 960 °C. SEM images were taken at the same magnification with a secondary electron detector.

Figure S10. CeO_2 NPs size distribution for Ce^{4+}-860 °C and Ce^{4+}-960 °C.

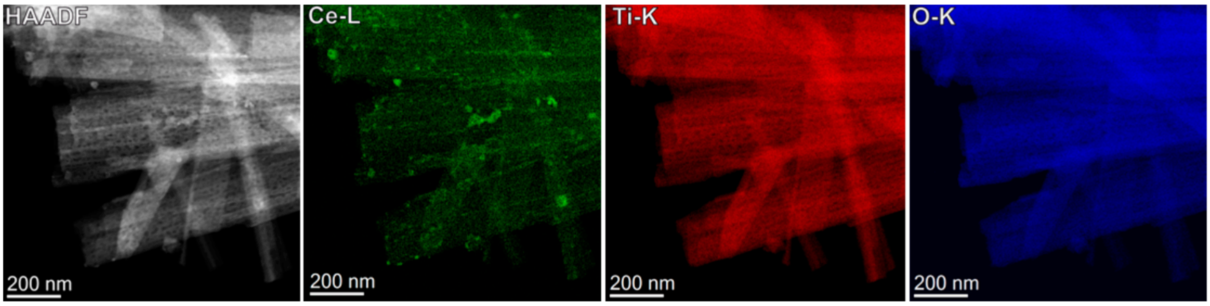

Figure S11. STEM-EDX elemental mapping of Ce^{4+}-620 °C.
